# Innate Lymphoid Cells in Helminth Infections—Obligatory or Accessory?

**DOI:** 10.3389/fimmu.2019.00620

**Published:** 2019-04-10

**Authors:** Stephan Löser, Katherine A. Smith, Rick M. Maizels

**Affiliations:** ^1^Wellcome Centre for Integrative Parasitology, Institute of Infection, Immunology and Inflammation, University of Glasgow, Glasgow, United Kingdom; ^2^Cardiff Institute of Infection and Immunity, Cardiff University, Cardiff, United Kingdom; ^3^Institute of Infectious Disease and Molecular Medicine, University of Cape Town, Cape Town, South Africa

**Keywords:** parasites, immunity, alarmin, cytokines, receptors

## Abstract

ILCs burst onto the immunological scene with their involvement in bacterial and helminth infections. As their influence has emerged, it has become clear that they play a fundamental role in regulating barrier tissue homeostasis and the immune response during inflammation. A subset of ILCs, ILC2s, has become the focus of attention for many helminth biologists—stepping into the limelight as both the elusive initiator and amplifier of the type-2 response. In many of the early reports, conclusions as to their function were based on experiments using unadapted parasites or immune-compromised hosts. In this review we re-examine the generation and function of type-2 ILCs in helminth infection and the extent to which their roles may be essential or redundant, in both primary and challenge infections. ILC2s will be discussed in terms of a broader innate network, which when in dialogue with adaptive immunity, allows the generation of the anti-parasite response. Finally, we will review how helminths manipulate ILC2 populations to benefit their survival, as well as dampen systemic inflammation in the host, and how this understanding may be used to improve strategies to control disease.

## Introduction

Parasitic helminth infections by tapeworms, roundworms or flukes present an ongoing threat to human health and quality of life, but also significantly dampen agricultural productivity through infection of livestock. Worldwide, over 2 billion people are infected with helminth parasites (1), and in the absence of any vaccines, control relies on repeated drug treatment. Understanding immunity to helminths is essential to develop new vaccines to eliminate these infections.

Until about a decade ago it was suggested that resistance to helminth infection relies predominantly on T helper 2 (Th2) cells of the adaptive immune system, orchestrating nematode expulsion in an antigen-specific manner. Since then a new player has entered the game, the type-2 innate lymphoid cells (ILC2), first discovered in T- and B-cell-deficient mice as a subset rapidly releasing type-2 cytokines, in response to stimulation by the alarmin IL-25 (2, 3).

Thereafter, convergent approaches, including functional testing of new IL-17 family members (3–5), determining innate sources of type-2 cytokines (6–8) and the characterization of mesenteric lymphoid clusters (9), all led to the identification of an innate lymphocyte population, capable of producing type-2 cytokines, albeit with varied monikers such as “nuocytes” (6) or “natural helper cells” (9). Soon re-branded as ILC2s, these innate cells were found localized to differing tissues and therefore primed to act as first responders to the immune challenges such as helminth infections before adaptive immunity has developed (10).

**Figure 1:**
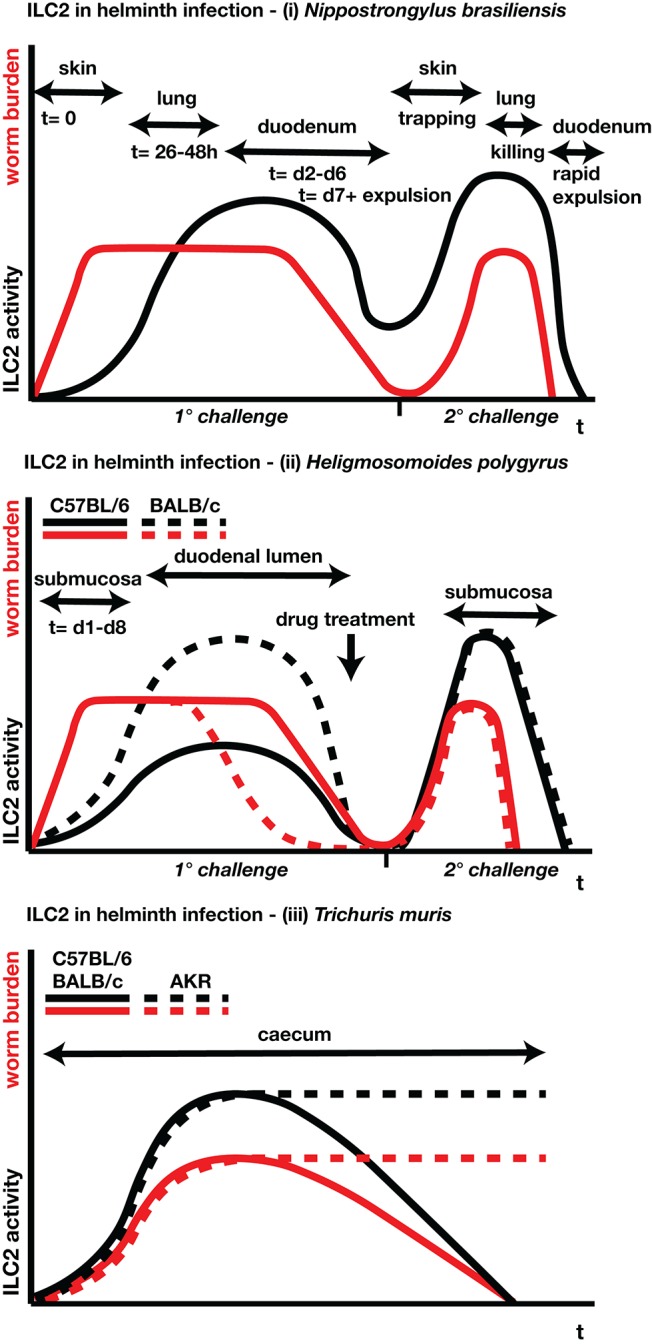
Schematic time-lines for parasite burdens and ILC2 responses for (i) *N. brasiliensis*, (ii) *H. polygyrus*, and (iii) *T. muris*. Red lines indicate parasite burdens (larval/adult numbers, egg production); black lines indicate ILC2 activity (proliferation, cytokine release). Location and timing of different parasite stages are indicated where appropriate. For *N. brasiliensis* and *H. polygyrus*, both primary and secondary infections are shown; primary *N. brasiliensis* infections are expelled naturally, while for *H. polygyrus* anthelmintic drug treatment is required in susceptible strains to clear the primary infection prior to a challenge re-infection. For *H. polygyrus* and *T. muris*, separate lines indicate genetically resistant and susceptible strains of mice.

Since the discovery of these innate Th2 cell surrogates, research delineating ILC2 functions, especially in the disease settings of helminth infection and allergic asthma, have dominated the scientific field of type-2 immunity. Wide-ranging studies have reported on the cells' origin and differentiation, plasticity, mobility, functionality and communication with various other cells of the immune system, and beyond (11, 12). Much has been learned from mouse models of intestinal helminth infection, in particular *Nippostrongylus brasiliensis*, a rat parasite which is poorly adapted (and thus readily dislodged) from mice, and 2 natural parasites of mice, *Heligmosomoides polygyrus* and *Trichuris muris* (Figure 1). ILC2s are also stimulated by helminths in the tissues, such as *Litomosoides sigmodontis* in the pleural cavity (13) and by the eggs of *Schistosoma mansoni* in the liver and lungs (14).

## Generation and Regulation of ILC2 Activation

In mice, the development of both ILC2s and Th2 cells starts with common lymphoid progenitors (CLPs) of the bone marrow (15, 16), but branches in two different directions. In fact, the development of all T lymphocytes begins with the differentiation of lymphoblasts from CLPs, their migration to the thymus, then if surviving both positive- and negative selection, they emerge as highly specific but antigen-naïve immune cells patrolling through the circulation, secondary lymphoid tissue and peripheral sites. Importantly, T cells only polarize into functional Th2 effector cells following specific antigen encounter in the presence of interleukin 4 (IL-4) (17).

In contrast, the development of ILC2s is rather primitive. CLPs give rise to common innate lymphoid progenitors (CILPs) dependent on the expression of various transcription factors, amongst others inhibitor of DNA binding 2 (Id2) and nuclear factor IL-3 induced (Nfil3) (9, 18, 19). Yang et al suggest that early innate lymphoid cell progenitors (EILPs) resemble an intermediate step in the differentiation of CLPs into CILPs. The development of this T cell factor-1 (TCF-1) positive population is still independent of Id2 and EILPs are progeny of both NK cells and ILCs (20). CILPs give rise to common helper innate lymphoid progenitors (CHILP) which differentiate based on promyelocytic leukemia zinc finger (Plzf) expression into ILC precursors (ILCPs) (21), able to polarize into three different innate lymphoid cell populations, ILC1s via expression of Tbx21/T-bet (22, 23), GATA3 positive ILC2s (15, 24) or, based on Rorγt expression, ILC3s (25, 26).

This broad classification of 3 ILC subsets may hide important distinctions within, for example, ILC2s which have been divided into natural and inflammatory, responsive to different cytokines (27), or between cells of similar cytokine profiles but different tissue residence, location or preference for skin, lung, or gut (28). ILC2s are mainly regarded as tissue–resident, expanding upon helminth infection at the respective mucosal site (29). This model has been challenged recently in a study demonstrating the migratory capabilities of IL-25-induced “inflammatory ILC2s” of the intestine (30, 31). These, when activated by IL-25, were found to enter the lymphatics and transfer to the lung where they contribute to the anti-helminth response. Controversy also arises over whether these ILC subsets are committed lineages, or show plasticity akin to their adaptive T cell cousins; while commitment would represent “trained immunity” in which the innate immune system is more prepared for subsequent encounters with the same pathogen, it might lock the innate response into an inappropriate and potentially pathogenic program that is difficult for the host to reverse.

## Helminths and ILC2s—Ancient Antagonists?

ILC2s and Th2 cells share common attributes, but they also differ in the complexity of their development, the rules governing their tissue tropism, and the specificity of immune stimuli to which they respond (30). This poses the question of whether the more complex and target-specific Th2 cells developed evolutionarily from ILC2s, or if both type-2 immune cell populations share vertical descent from one ancestral population. The first would imply that ILC2s emerged prior to Th2 cells, potentially as an elementary cell type to combat helminth infection in ancient hosts.

To shed light on the evolutionary development of the three different ILC populations, including ILC2s, Vivier et al analyzed comparative expression of critical genes in different vertebrate species from the lamprey to mammals (32). Notably, a significant part of the ILC progenitor gene profile, including *Id2, Nfil3* and *Gata3*, as well as parts of the ILC2 profile, including *Ptpn13, Ar, Rxrg, Ccr8*, and *Hs3st1* are already evident in jawless vertebrates, whereas the *Gata3*-controlled type-2 cytokine genes, *Il5* and *Il13*, crucial for ILC2 and Th2 cell effector functions in mice and humans, were exclusively found in birds and mammals (32).

This initial study certainly suggests that ILC2s emerged in evolution of the earliest agnathan vertebrates, in response to helminth parasites. Even today, sea lamprey intestines contain a variety of nematode species, ingested while feeding on fish (33). A different perspective on the evolutionary development comes from a phylogenetic analysis of genes crucial for ILC2 activation in mammals. Here it has been highlighted that lampreys do express IL-17 family orthologs, amongst them IL-25 (34). Orthologs of the crucial receptor heterodimer subunit IL-17RB for IL-25 signaling in humans and mice however, have been detected only from the emergence of cartilaginous fish onwards and have not been found in jawless fish (34).

Phylogenetic analyses of genes involved in the differentiation of different immune cell populations suggest that ILC2s, as T-cells, developed in ancient mammalian ancestors. Substantial differences in the differentiation of both cell types certainly suggest that their relationship is distinct and that ILC2s are closely related to the other ILC subtypes. A different perspective might be provided through comparisons of genome-wide chromatin accessibility of both populations during helminth infection (35). Hierarchical clustering of these regulatory ensembles—termed “regulomes” within Th2 cells and ILC2s isolated from lungs at day 10 post *N. brasiliensis* infection confirmed the difference between pre-established ILC2s and naïve CD4 T cells. Regulomes of both cells during infection showed high similarity, not discounting a potential close relationship between both cell types during evolution and more ancient ILC ancestor that has preceded Th2 cells (35).

This perspective is further supported by the aforementioned study on the migration of activated iILC2s, highlighting that both T cells and iILC2s share the capability to migrate along a sphingosine-1-phosphate (S1P) gradient, a mechanism crucial for effector T cells to leave lymphoid tissues and essential for iILC2s to enter the lymphatics from the intestine (31). The report extends the parallelism of both type-2 cell types beyond the level of genomics and immune effector functions not excluding that the S1P trafficking mechanism of T cells was adopted from ancient ILCs (30).

## Alarmin Activation of ILC2s—the Role of IL-25

As discussed above, ILC2 development requires the transcription factor GATA3 (24) and the nuclear receptor RORα (15, 36). Once differentiated, ILC2s are highly responsive to the alarmins, IL-25, IL-33 and TSLP, binding, respectively to the heterodimeric receptors IL17RA/IL17RB, ST2/IL1RacP, and TSLPR(CRLF2)/IL7Rα (37), while also requiring signaling through the common cytokine receptor γ chain (γ_c_) that is shared by IL-2, IL-4, IL-7, IL-9, IL-15, and IL-21 (9).

IL-25 is closely linked, in presence and function, with helminth infections, and was first characterized by elevated expression in the small intestine following *N. brasiliensis* infection, leading to IL-5 production by the then unnamed ILC2s (3). IL-25^−/−^ mice were then shown to be unable to expel *T. muris*, while genetically susceptible mice given exogenous IL-25 became resistant to infection (38). Similarly, IL-25^−/−^ mice were used to demonstrate that IL-25-dependent activation of ILC2s is required for protective immunity to *N. brasiliensis* infection (4). Moreover, it was demonstrated that transfer of IL-25-stimulated ILCs could mediate expulsion of *N. brasiliensis* in mouse strains lacking key type-2 components, such as IL-13, IL-25, or IL-33 (6). In particular, while exogenous IL-25 induced worm clearance in RAG-deficient mice (31), it did not do so in animals lacking both adaptive immunity and ILCs (Rag-2^−/−^ x $\gamma_{\text{c}}^{-/-}$ mice) (7). This latter study also demonstrated that transfer of ILCs alone was not able to induce worm clearance in Rag-2^−/−^ x $\gamma_{\text{c}}^{-/-}$ mice unless exogenous IL-25 was administered to recipient animals.

Subsequently, IL-25 has been shown to activate a variety of innate cells, including multi-potent progenitor type-2 cells (39), NKT cells (40, 41) as well as monocytes and eosinophils (42). Within the ILC2 population, it has been proposed that one subset of “inflammatory ILC2s” (iILC2s) preferentially express the IL-25 receptor, and not the IL-33 receptor ST2 (27). Inflammatory ILC2s arise early in *N. brasiliensis* infection and give rise to IL-33-responsive “natural ILC2s” for worm expulsion, although it was also found that iILC2s, co-expressing GATA3, and RORγt, have the capacity to switch to IL-17 producing ILC3s in the different setting of fungal infection (27).

**Figure 2:**
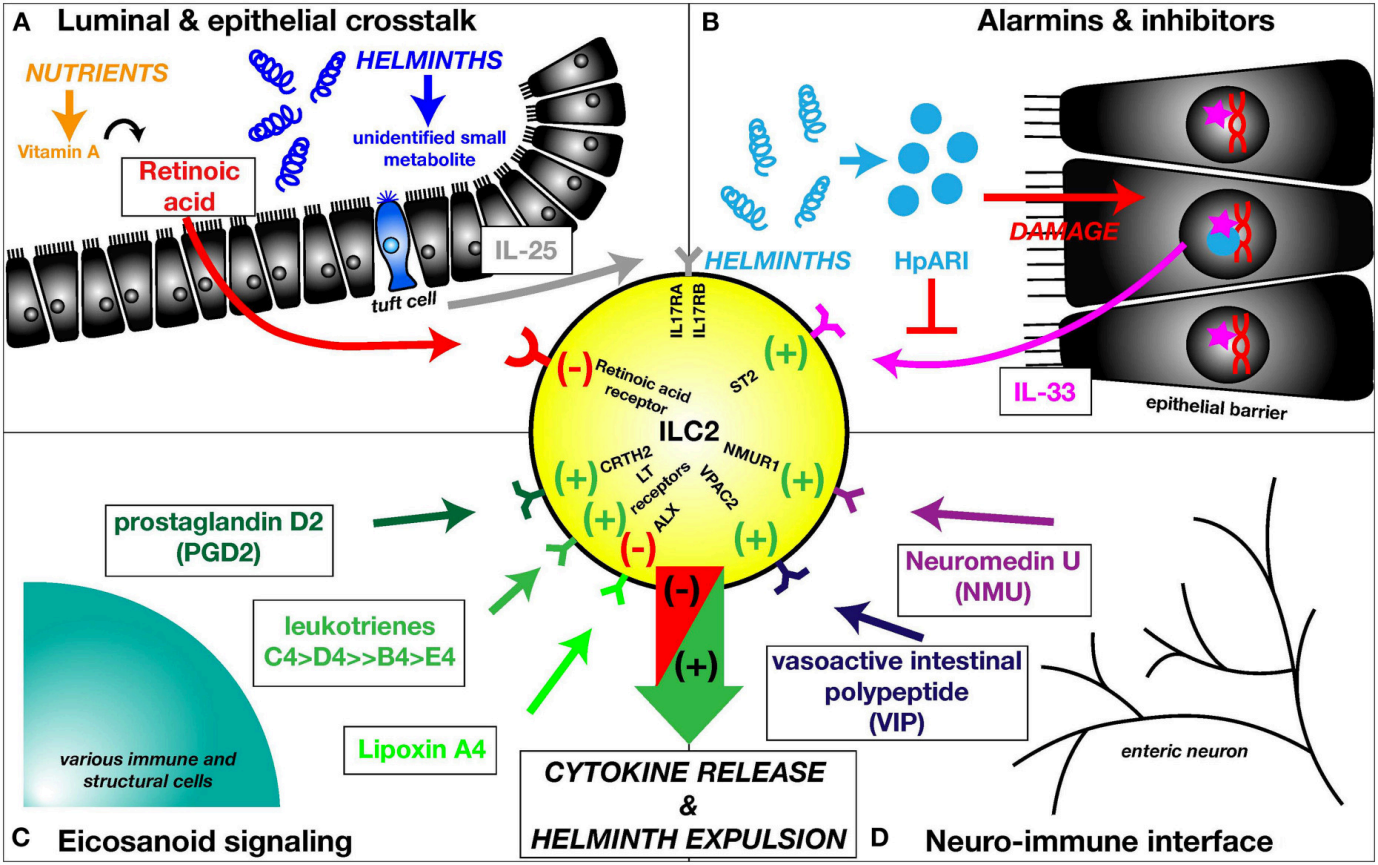
ILC2s are embedded into a multifaceted network of barrier- and neuro-immune responses. **(A)** *Luminal & epithelial crosstalk*. Intestinal nematodes are detected by tuft cells of the small intestinal epithelium. While the helminth-derived mediator activating tuft cells is unknown, these chemosensory cells release IL-25, activating the IL-25 receptor (IL17RA/IL17RB) on ILC2s. ILC2s are negatively regulated by the vitamin A metabolite retinoic acid, signaling through retinoic acid receptors. **(B)** *Alarmins & inhibitors*. In addition to IL-25, ILC2s are induced by IL-33, an alarmin located inside the nuclei of epithelial cells. Upon damage to the epithelial lining, IL-33 is released. The nematode-derived alarmin inhibitor HpARI is able to sequester IL-33 inside the nuclei of damaged cells, attenuating the IL-33-induced ILC2 activation. **(C)** *Eicosanoid signaling*. Additionally, ILC2s are activated by various eicosanoids, including PGD2 and leukotrienes, activating the respective receptors, while negative regulation of ILC2s has been documented for lipoxin A4. **(D)** *Neuro-immune interface*. ILC2s are essential for the communication between enteric neurons and the epithelial barrier. NMU, released by neurons during helminth infections activates ILC2s via NMUR1 potentiating the epithelial response initiated to discharge the intruders. ILC2s are also stimulated by VIP, but VIP release by neurons during helminth infection has not been documented as of yet.

Recently, it was discovered that epithelial tuft cells of the small intestine detect the presence of helminths and release IL-25 (43–45), resulting in ILC2 proliferation (44) (Figure 2A). While the helminth derived mediator responsible for activating the tuft cell signaling cascade is still unknown, it is clear that protozoans are capable to induce tuft cells and successively ILC2 activation, through the release of succinate and stimulation of the succinate receptor GPR91 (46, 47). The activation of intestinal ILC2s by IL-25 is negatively regulated by the deubiquitinase A20 (*Tnfaip3*), which binds and inhibits the IL-25 receptor subunit IL-17RA (48). During infection with *N. brasiliensis*, A20 expression in ILC2s is downregulated, allowing increased pathogen-induced ILC2 proliferation (49).

While two other key alarmin cytokines, IL-33, discussed below, and thymic stromal lymphopoietin (TSLP) are able to induce ILC2 expansion, the responsiveness to these differs between ILC2 populations located at various mucosal sites. Small intestinal ILC2s express elevated levels of IL-17RB and are therefore responsive to tuft cell-derived IL-25, whereas pulmonary and adipose tissue ILC2s express high levels of ST2, resulting in increased IL-33 sensitivity (50). While not essential for ILC differentiation, TSLP is particularly important for activating ILC2s in the skin, for example in models of atopic dermatitis, which is abated in *Tslpr*^−/−^ mice (51) and is also required for immunity to *T. muris* in the intestinal tract (52). However, TSLP also activates dendritic cells to promote adaptive Th2 responses (53), and acts on other innate populations such as basophils (54), so that it promotes type 2 responses at multiple and complementary levels.

## IL-33, Key Player for ILC2s

IL-33, an IL-1-like cytokine that potently activates the type-2 response, is released from damaged epithelial cells (Figure 2B), and from innate subsets such as mast cells responding to damage signals from their environment (55–57). The role of IL-33 in ILC2 activation has been explored intensively by utilizing murine helminth models with pulmonary life cycle stages, including *N. brasiliensis* and the threadworm *Strongyloides venezuelensis*. Both induce a significant increase in lung/airway IL-33, IL-33 dependent ILC2 activation, type-2 cytokine release and eosinophilia (6, 7, 9, 58, 59). IL-33-deficient mice show a significantly elevated intestinal *N. brasiliensis* worm burden at day 6 p.i. and increased fecal egg counts at day 8 post *S. venezuelensis* infection, underlining a contribution of IL-33 activated ILC2s to the clearance of these nematodes (58, 59). Mice lacking the IL-33-specific receptor subunit ST2 are also highly susceptible to *H. polygyrus* infection (60), in addition, exogenous IL-33 confers resistance on mice genetically susceptible to *T. muris* (61).

As deficiency in either IL-25 or IL-33 results in greatly enhanced susceptibility to helminth infection, these alarmins are not generally redundant. However, it is possible to circumvent the lack of one alarmin with cell populations strongly activated by another. Thus, transfer of ILC2s cultured in the presence of IL-7 and IL-33 restored expulsion of *N. brasiliensis* in mice lacking the receptor for IL-25 (*Il17rb*^−/^^−^) (6). Moreover, the same authors reported that transfer of IL-13-expressing ILCs was sufficient to induce expulsion in mice lacking both receptors for IL-25 and IL33 (*Il17rb/Il1rl1*^−/−^) or type-2 cytokines (*Il-4/Il-13*^−/^^−^). Here, as discussed below, the ability of ILC2s to mediate worm expulsion was dependent on dialogue with the adaptive immune system and generation of antigen-specific T cell production of IL-13.

## ILC2s—Other Signals, Small and Large

A range of small molecules are also able to orchestrate ILC2 functions, including arachadonic acid-derived eicosanoid (20-carbon) lipids. Human ILC2s can be activated by prostaglandin D2 through the CRTH2 (DP2) receptor (Figure 2C) (62) and their cytokine production can be further enhanced by stimulation with leukotriene E4 (63). In mice, leukotrienes can sufficiently induce type-2 cytokine production by ILC2s, with leukotrienes C4 and D4 being most efficient in provoking this response, whereas stimulation with leukotriene B4 and E4 only resulted in marginal IL-13 production (Figure 2C) (64, 65). In particular, leukotriene D4 drives IL-4 production by ILCs (66, 67). The role of eicosanoid mediators in nematode infection was investigated using gene-deficient mice of the respective leukotriene receptors or synthases, highlighting that deficiency in the LTB4 high affinity receptor *Ltb4r1* did not affect ILC2 activation in the lungs of *N. brasiliensis* infected mice. Activation was reduced by *Cystlt1r* (LTD4/LTC4 receptor) or *Ltc4s* (LTC4 synthase) deletion and abrogation of ILC2 activation was achieved in mice lacking *Alox5* (5 lipoxygenase, which catalyses the initial step of leukotriene synthesis) or the combination of *Ltb4r1* and *Ltc4s* (65). Inhibitory properties with regards to ILC2 activation were reported for lipoxin A4 (Figure 2C) (68) and prostaglandin E2 for human (69) and prostaglandin I2 for mouse (70) ILC2 cells.

Recent studies have highlighted the sensitivity of ILC2s to dietary vitamin A deficiency (71) and related their function to a requirement for fatty acids, acquired from the environment (72). In the event of vitamin A deficiency, the subsequent absence of retinoic acid resulted in the predominance of ILC2s in the small intestine and an increased resistance to *T. muris* infection (Figure 2A) (71). Under these conditions, ILC2s in the mesenteric adipose tissue increased their acquisition of long-chain fatty acids and raised production of IL-5 and IL-13 (72). Thus, as a result of nutrient deprivation, enhanced ILC2 activity is allowed ensuring that helminths are discharged more efficiently.

More recently, the A2B adenosine receptor was found to promote ILC2 activation and type-2 cytokine expression, controlling the primary and secondary immune response to *H. polygyrus* infection and the primary immune response to *N. brasiliensis* infection (73). This study also revealed adenosine as a novel danger-associated molecular pattern (DAMP), responsible for initiating helminth-induced type-2 responses, through activation of ILC2s.

Additional receptors reported to be highly expressed by ILC2s and required for their expansion and function include the tumor necrosis factor (TNF)-receptor superfamily member DR3 (TNFRSF25) and the IL-9R. In the absence of TL1A ligation to DR3, there is significantly reduced ILC2 expansion, type-2 cytokine production and *N. brasiliensis* expulsion (74), whereas IL-9R signaling was required for ILC2 accumulation, expulsion of *N. brasiliensis* and resolution of tissue damage in the lung (75). IL-4- and IL-13-dependent expression of the acidic mammalian chitinase (AMCase) was also found to promote ILC2 expansion in the lung and expulsion of both *N. brasiliensis* and *H. polygyrus* from the gut (76).

Recently, the capacity of ILC2s to migrate between mucosal sites has become recognized with the experimental demonstration of a S1P-dependent pathway leading ILCs from the gut to the lung (30). MLN and lung iILC2s express S1P receptors and FTY720 inhibition of this pathway blocked the accumulation of intestinal iILC2s in the lung. When administered to Rag1^−/−^ mice, FTY720 induced a significant increase in mortality to *N. brasiliensis* infection, which in turn can be prevented by an intravenous adoptive transfer of iILC2s prior to FTY720 administration (31). The role of chemokines in ILC2 migration and trafficking is understudied, though gene expression studies show that in comparison to other NK-cell and ILC subsets, small intestinal ILC2s express enhanced levels of *Cxcr6* and *Ccr4, 8*, and *9*, while also producing attenuated levels of the chemokine ligand *Ccl1* (77). Additionally, ILC2s express a number of surface receptors regulating cell-cell interactions. ILC2s express KLRG1, which as shown using human skin ILC2s, can interact with E-cadherin and dampen ILC2 specific type-2 cytokine expression (78). ILC2s also display ligands for OX40 and ICOS that mediate interactions with T cells as discussed below.

### ILC2s at the Neuro-Immune Interface

Most recently, emphasis has been placed on delineating ILC2 function in the neuro-immune network during helminth infection. ILC2s are located in close proximity to enteric neurons, primed to receive neuronal signals during infection. ILC2s were found to selectively express Neuromedin U receptor 1 (*Nmur1*), whereas mucosal neurons expressed the small neuropeptide neuromedin U (*Nmu*) (79, 80). Following infection with *N. brasiliensis, Nmu* expression was increased in both the lung and the gut, resulting in robust ILC2 responses through *Nmur1* and worm clearance (Figure 2D); in contrast, if adoptively transferred into *Rag2 Il2rg* deficient mice, *Nmur1* (NMU receptor 1) deficient ILC2s produce significantly reduced amounts of type-2 cytokine than their gene sufficient counterparts (79, 80). ILC2s are also activated by the neuropeptides vasoactive intestinal peptide (VIP) (81) (Figure 2D) and calcitonin gene-related peptide (CGRP) (82), although as yet it is unclear if these neuronal effectors play a significant role in ILC2 activation during helminth infection.

Interestingly, ILC2 activity was reported to be negatively regulated by acetylcholine through the nicotinic acetylcholine receptor α7nAChR (83), catecholamines (e.g., epinephrine) ligating the β2-adrenergic receptor (84), as well as the aryl hydrocarbon receptor (AhR) although in the latter case this may operate through preferential expansion of ILC3 cells (85).

## Dialogue With Adaptive Immunity

An important early finding in the field was that ILCs did not prosper when transferred to RAG-deficient animals, and indeed ILC2 transfer did not succeed in conferring immunity to *N. brasiliensis* to this genotype (6). It is now well appreciated that ILC2s closely communicate with the adaptive immune system, allowing for the initiation and amplification of a robust type-2 response (86). For example, ILC2s promote Th2 immunity by enhancing CD4^+^ T cell function through MHCII expression (87, 88). In the setting of helminth infection, ILC2s express high levels of surface MHC class II, as well as IL-25R (IL-17RB), and drive Th2 and Th9-mediated clearance of *Trichinella spiralis* (89). ILC2 “help” for Th2 cell differentiation may require IL-4, as mice lacking this gene only in the ILC compartment show attenuated Th2 cytokine production following *H. polygyrus* infection, although no increase in susceptibility was reported (67).

ILC2s have also been shown to upregulate expression of the checkpoint inhibitor PD-L1, following infection with *N. brasiliensis*. Surprisingly, expression of this molecule by ILC2s promoted production of IL-13 by Th2 cells and enhanced expulsion of the gastrointestinal nematode (90). Interestingly, ILC2s were also reported to promote regulatory T-cell expansion following *N. brasiliensis* infection. This interaction was suggested to be reliant on the expression of ICOSL by ILC2s and ICOS by Tregs (91). In the context of helminth infection with *N. brasiliensis*, lung ILC2s express OX40L in response to IL-33 stimulation allowing enhanced communication with OX40^+^ T-cells (92). ILC-specific OX40L deficient *Il7r(Cre) Tnfsf4(fl/fl)* mice display a suppressed adaptive type-2 immune profile at d28 p.i., with significantly decreased numbers of both GATA3^±^ Tregs and GATA3^+^ Th2 cells, together with a mildly elevated intestinal worm burden at day 5 p.i. (92). Finally, in addition to ICOSL, ILC2s express ICOS, which can act as an inhibitory receptor in interactions with regulatory T cells (93).

## ILC2s in Helminth Infections—Sufficiency, Necessity, or Redundancy?

The immune system has evolved multiple protection mechanisms against helminth infection, and the literature has documented a range of cell types, which if suitably activated and expanded, can mediate helminth expulsion (94, 95). For reasons discussed above, elimination of *N. brasiliensis* may be achieved more readily by multiple mechanisms, providing a sensitive test-bed for immunity to helminths. In this system, the principle has been established that innate sources of IL-4/IL-13 are sufficient for expulsion in animals unable to produce these cytokines within the T cell population (96). Consistent with this, adoptive transfer of ILC2s (6) induces nematode expulsion. However, transgenic mice with chronic eosinophilia [through IL-5 over-expression (97)] are also resistant to infection. The demonstration that disparate cell types are individually sufficient to combat helminth infection in mouse models is consonant with other studies showing that few are essential; demonstrating considerable redundancy.

In mouse models other than *N. brasiliensis*, with parasite species naturally adapted to the mouse, adoptive cell transfer of ILC2s has not been quite so effective, inducing partial, but not complete, clearance of *T. muris* (5) and *H. polygyrus* (67). In the case of *H. polygyrus*, Hepworth and colleagues (98) reported “negligible” MLN ILC2 numbers (0.2 × 10^5^ cells) at day 6 of infection of C57BL/6 mice, requiring exogenous IL-25 treatment to boost the number of MLN ILC2 cells to reach levels observed during *N. brasiliensis* infection (*H. polygyrus*: 3 × 10^5^ cells vs. *N. brasiliensis*: 5 × 10^5^ at d5 p.i.) (99). This may reflect the poor responsiveness of this strain, as ILC2 responses are greater in more resistant mouse strains such as BALB/c and SJL (100) (Figure 1B). However, the theme of multiple pathways to resistance recurs with *H. polygyrus*, as both transfer of M2 macrophages (101) or transgenic amplification of mast cells (resulting in higher ILC2 levels) (57) are protective against infection.

ILC2-mediated immunity to *N. brasiliensis* infection can also be driven by other helminths, such as *S. venezuelensis*, which evokes a sufficiently strong expansion of ILCs in the lung, that a subsequent *N. brasiliensis* infection is significantly reduced in both worm number and egg output (102). Consistent with other information on the lung environment, the ILC2 response at this site required IL-33. Perhaps this finding recalls an ancient evolutionary role of ILCs in providing early chordates with generic protection against helminth infection, as discussed above.

An important point of consideration is that ILC2s are not the sole target of IL-25 activation in helminth infection, given evidence that other innate cell types such as monocytes (101), other myeloid cells (103) and NKT cells are responsive to this cytokine. In addition, IL-25 promotes progenitor cells [mpp-type2, (5)], which can differentiate into mast cells. Many of these cell types are thought to be involved in immunity to helminth infection. Indeed, utilizing the *H. polygyrus* model of nematode infection, we were able to show that worm expulsion could be induced by IL-4 and IL-25 in RAG-deficient mice, and that this process was unimpeded by depletion of CD90^+^ ILCs prior to cytokine administration (42). Hence in these more tenacious helminth infections, ILC2s may not be sufficient for immunity, and indeed may even be redundant.

## ILCs in Humans

While these studies extended our appreciation of ILC2s across a considerable number of helminth infections in mice, less information is available on their involvement in human helminth infections (104, 105). The theme of redundancy is echoed in human studies, as individuals lacking ILCs (following hematopoietic stem cell transplantation therapy) appear to lead healthy lives for many years (106, 107), although these patients are not likely to have been exposed to helminth parasite infections.

As highlighted by Nausch and Mutapi, many helminth parasites invade through, or establish a niche in, the skin of their host, and consequently are likely to encounter skin ILC2 cells (105). Parasites also frequently interact with mucosal sites, in particular the gastrointestinal tract, where ILC2s may be activated. Due to inaccessibility of these tissues in infected patients, studies delineating the immunology of human nematode infections are mostly reduced to analyses of peripheral blood, in which ILC2s may represent < 0.05% of live blood leucocytes (105). Nonetheless, helminths do cause changes in peripheral blood ILC populations. In a patient cohort of Zimbabwean children infected with *S. haematobium*, significantly fewer circulating ILCs were found in 6–13 years-olds, but normal levels observed in Schistosome-specific antibody positive infected children older than 14 years (108). Thus, there is an age-specific effect, which speaks to the increased relevance of ILC2s during early life before immunity is dominated by a mature adaptive immune response. In a second study, blood was taken from adult patients infected with the filarial nematodes *Loa loa, O. volvulus*, and *Wuchereria bancrofti*, and ILC2s and ILC3s were analyzed together as CD127^+^CD117^+^ cells (13). These authors reported increased blood ILCs in these infections, contrasting with the observations made of *S. haematobium* infection, highlighting that the ILC response might be heterogeneous in different infection settings and age groups.

The inaccessibility of ILC2s in the key tissues of humans returns the focus to events induced by human parasites in mouse models of helminth infection. Initial studies highlight an upregulation of key ILC2- inducing alarmin genes *Il1a, Il1b, Il33*, and *Tslp* shortly (6 h) after percutaneous *S. mansoni* cercariae infection of the pinna (109). Further, a study of combined IL-25, IL-33, and TSLP cytokine targeting during chronic murine *S. mansoni* infection revealed that ablation of either or both IL-25 and IL-33 is not sufficient to significantly alter *S. mansoni*-induced type-2 pathology in both the lung and the liver (110). Indeed it was needed to target all 3 alarmins utilizing IL-33^−/−^ x TSLP^−/−^ mice administered anti-IL-25 from week 4 to significantly reduce the liver granuloma volume at week 9, concomitant with a reduced granuloma eosinophil percentage and attenuated MLN IL13^+^ ILC2 levels. However, by week 12 alarmin neutralization did not alter type-2 pathology, and Th2 cytokines had actually increased (110), again underlining that ILC2 effector functions may be restricted to the acute phase of type-2 immunity and that loss of ILC2 function can be compensated for by accelerated activity of Th2 cells.

## Can Helminths Manipulate ILC2 Function?

Helminth infections can clearly stimulate type-2 responses yet have the ability to modify them, as part of their immune evasion strategy. An obvious immune cell candidate for targeting in order to avoid effective immunity is the ILC2. Following infection with the chronic helminth parasite *H. polygyrus*, there is a limited expansion of ILC2s in the mesenteric lymph nodes (MLN) (42, 98, 100). Indeed, a recent study revealed preferential trafficking of LTi-like ILC3s to the MLN following *H. polygyrus* infection (111). Infection with this chronic gastrointestinal nematode has been shown to promote the release of host-derived IL-1β, which limits IL-25 production and the subsequent activation of ILC2s (112).

Further evidence of the ability of *H. polygyrus* in adapting to the hosts' capacity to drive ILC2 activation via alarmin cytokines was recently revealed by the discovery of the IL-33 inhibitor HpARI, which is released by the helminth and sequesters IL-33 in the tissue (Figure 2B) (113). In addition, the same parasite releases extracellular vesicles that target expression of ST2, the IL-33 receptor, and are able to dampen the ILC2 response to *Alternaria alternata* fungal allergen challenge *in vivo* (114). Many helminths, *H. polygyrus* included, further modulate the adaptive immune system by promoting regulatory population such as regulatory T cells (115); with the recent description of IL-10-producing regulatory ILCs (116), it will be interesting to establish if some parasite species can dampen host immunity through immunosuppressive cells of the innate, as well as the adaptive, immune system.

## Conclusion

ILC2s are clearly an inherent feature of the immune response to helminth infection, and in all probability their evolution has been driven by the threat of parasites. While in experimental model systems they are not always found to be essential, they are often center stage, particularly in the early phases of infection of each helminth system so far analyzed. They also form an important conceptual and mechanistic link with the allergic response that will allow us to understand in more detail the genesis and control of allergic disorders. In this respect, a much fuller analysis of ILC biology and function in the human type 2 response, both in helminth infections and allergy, is eagerly awaited. Finally, the question remains to be answered of whether we can design new interventions, ideally vaccines, which take advantage of the ILC2 phenotype to promote protective immunity against helminth parasites and to control and remove the enormous burden of worm infections across the world.

## Author Contributions

All authors listed have made a substantial, direct and intellectual contribution to the work, and approved it for publication. SL, KS, and RM have planned, written, and revised the article collaboratively. SL and RM have planned and created the figures.

### Conflict of Interest Statement

The authors declare that the research was conducted in the absence of any commercial or financial relationships that could be construed as a potential conflict of interest.
